# PIPER adult comfort: an open-source full body human body model for seating comfort assessment and its validation under static loading conditions

**DOI:** 10.3389/fbioe.2023.1170768

**Published:** 2023-05-30

**Authors:** Shenghui Liu, Philippe Beillas, Li Ding, Xuguang Wang

**Affiliations:** ^1^ Key Laboratory of Biomechanics and Mechanobiology, Ministry of Education, Beijing Advanced Innovation Center for Biomedical Engineering, School of Biological Science and Medical Engineering, Beihang University, Beijing, China; ^2^ Université de Lyon, Université Claude Bernard Lyon 1, Université Gustave Eiffel, LBMC UMR_T 9406, Lyon, France

**Keywords:** human body model, finite elements, seating comfort/discomfort, validation, pressure/force distribution, open source

## Abstract

**Introduction:** In this paper we introduce an adult-sized FE full-body HBM for seating comfort assessments and present its validation in different static seating conditions in terms of pressure distribution and contact forces.

**Methods:** We morphed the PIPER Child model into a male adult-sized model with the help of different target sources including his body surface scans, and spinal and pelvic bone surfaces and an open sourced full body skeleton. We also introduced soft tissue sliding under the ischial tuberosities (ITs). The initial model was adapted for seating applications with low modulus soft tissue material property and mesh refinements for buttock regions, etc. We compared the contact forces and pressure-related parameters simulated using the adult HBM with those obtained experimentally from the person whose data was used for the model development. Four seat configurations, with the seat pan angle varying from 0° to 15° and seat-to-back angle fixed at 100°, were tested.

**Results:** The adult HBM could correctly simulate the contact forces on the backrest, seat pan, and foot support with an average error of less than 22.3 N and 15.5 N in the horizontal and vertical directions, which is small considering the body weight (785 N). In terms of contact area, peak, and mean pressure, the simulation matched well with the experiment for the seat pan. With soft tissue sliding, higher soft tissue compression was obtained in agreement with the observations from recent MRI studies.

**Discussion:** The present adult model could be used as a reference using a morphing tool as proposed in PIPER. The model will be published openly online as part of the PIPER open-source project (www.PIPER-project.org) to facilitate its reuse and improvement as well as its specific adaptation for different applications.

## 1 Introduction

Sustained loads on the soft tissue of the buttocks may cause seating discomfort or even physiological problems such as pressure ulcers (Gefen, 2008). Along with pressure, soft tissue deformations and internal loading in terms of compression, strain, and stress are generally considered relevant for seating discomfort assessment (De Looze et al., 2003). However, these internal biomechanical parameters cannot be directly measured *in vivo*. With the development of computational capability, different biomechanical models such as finite element (FE) models (Verver et al., 2004; Siefert et al., 2008; Grujicic et al., 2009; Al-Dirini et al., 2016; Beaugonin and Borot, 2019), multibody modeling with (Rasmussen et al., 2009; Grujicic et al., 2009) and without (Campos and Xi, 2020; Zhong et al., 2022) considering musculoskeletal systems are built for seating dis/comfort assessment or seat design. To estimate the internal biomechanical parameters such as soft tissue deformation, deformable finite element (FE) human body models (HBM) are required (see a review by Savonnet et al., 2018). There are mainly two types of FE models: partial thigh-buttocks models built from medical images (Verver et al., 2004; Al-Dirini et al., 2016; Moerman et al., 2016; Cheng et al., 2018; Macron et al., 2018), and full-body models from medical images for skeleton and 3D body scans for skin geometry (Siefert et al., 2008; Grujicic et al., 2009; Du et al., 2013; Huang et al., 2015; Lazarov et al., 2015; Beaugonin and Borot, 2019; Huang et al., 2022). The partial models can only be used for evaluating the seat pan, and boundary conditions such as external contact forces and position of the bones cannot be easily defined under real seating conditions. To evaluate the comfort of a complete seat, a full human body model (HBM) is needed. A few HBMs were proposed by some researchers (Grujicic et al., 2009; Du et al., 2013; Huang et al., 2015; Huang et al., 2022) as well as by some engineering software companies (Siefert et al., 2008; Beaugonin and Borot, 2019). However, for both existing partial and whole-body models, the validation was performed in comparison with pressure-related parameters under either very simplified conditions or a single specific seat condition. For instance, Huang et al. (2015) developed a HBM based on the Hybrid III 50th Percentile Male dummy to evaluate the comfort and design of automotive driving seats. They only used a rigid chair with two flat rectangular plates representing the seat pan and backrest to validate their model. Without considering soft cushion, human body interaction with the seat cannot be correctly simulated. Grujicic et al. (2009) validated their full HBM with respect to the measured pressure distribution reported by Verver et al. (2004) based on a male subject sitting on a sitting on a seat (rigid or with a soft cushion) with the feet unsupported. When using experimental data from a third party, personalizing an HBM is challenging, as the test subject geometry is typically not available. This makes comparisons between simulation and experiment difficult to interpret if the model’s geometry and mass distribution deviate from the test subject. Also, the test conditions were very simplified and different from real seating conditions. In a more recent study looking at the comfort of seat cushion, Yadav et al. (2021) validated their buttock-thigh partial FE model simplified from the 50th male full body model from the Global Human Body Model Consortium against the pressures. Twenty participants were asked to sit on different cushions put on a flat table. The participant’s feet and back were unsupported. Again, the test conditions were over-simplified, and the corresponding loadings were likely unrealistic. Moreover, seat configurations vary in real life depending on use conditions (e.g., at the office or during transportation). To assess the effect of seat design, an HBM should also be validated under more realistic seating conditions covering a large range of existing seat configurations. As the HBMs give the possibility of estimating internal loading, it is also desirable to verify the soft tissue compression as well, especially under the ischial tuberosities (ITs). As far as we know, none of the existing models simulated the soft tissue displacement away from the ischium once seated, which was observed with an open MRI system in recent years by several researchers (Sonenblum et al., 2013; Sonenblum et al., 2015; Sonenblum et al., 2018; Wang et al., 2021). More importantly, a human model should be developed within an identified application context. Application specific adaptation of the model is often needed. Existing HBM models for seating comfort evaluation are not open source, thus limiting access to model adaptations, improvements, and comparisons with other models. Therefore, we believe that there is a need to propose an open source HBM, which should be validated under real seating conditions for seating comfort application.

A seat is designed to accommodate multiple body sized persons. As a first step to provide a virtual seating assessment tool, we recently morphed the PIPER open source child model (PIPER Child model), initially developed for impact simulation (www.PIPER-project.org), into an adult male model (Liu et al., 2020). This paper will present how the morphed adult model was further adapted and validated using the experimental data collected from the same adult male on a reconfigurable seat. The choice of a subject specific modelling approach was done to enable the comparison of measurements without the bias of geometrical and mass differences. Both shear and normal components of contact forces, and pressure parameters, such as contact area, peak pressure, and pressure profiles, will be compared between FE simulations and experimental results. In addition, we will preliminarily analyze the effects of soft tissue sliding on soft tissue compression under the ITs. Beyond this work, other body sized models could be generated using existing personalizing tools such as the PIPER ones (www.PIPER-project.org).

This paper will first present the adult-sized FE full-body HBM, then the experimental data used for validation and finally the comparison between simulations and experimental observations. The limitations of the model and its future improvements will also be discussed.

## 2 Development of an adult male HBM for comfort

### 2.1 Model development and adaptions

**FIGURE 1:**
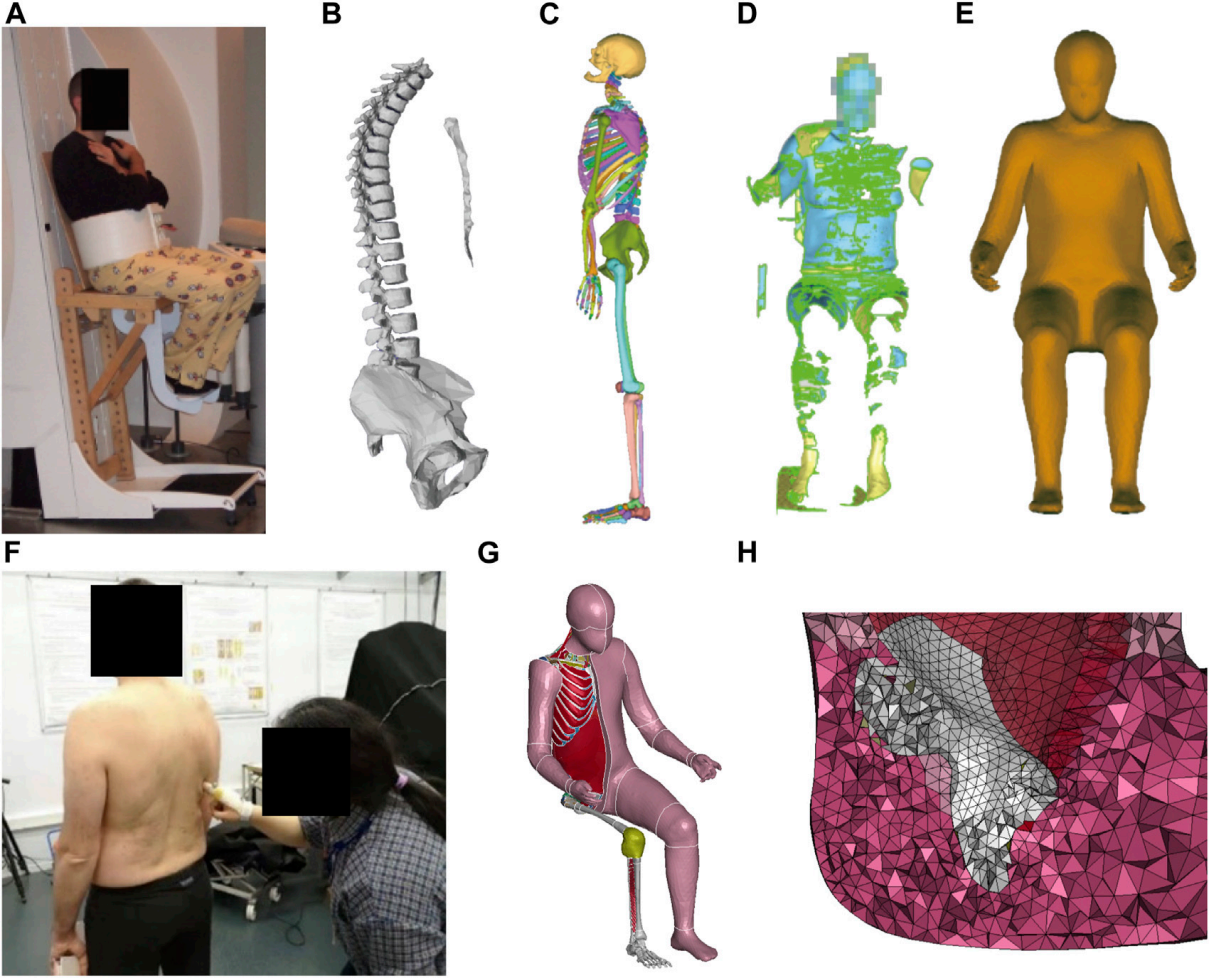
Data sources for model development: **(A)** Experimental seating set-up—used in the original MRI study by Beillas et al. (2009)—was reused for the body scan, **(B)** Partial skeleton of the target subject in the target sitting posture from Beillas et al. (2009), **(C)** Full skeleton in supine posture of the subject LTE605 from the open PIPER datasets (www.PIPER-project.org), **(D)** Body scan in the same sitting posture as in **(A)**, **(E)** Processed target skin surface in the target position after smoothing and anonymizing, **(F)** Palpating the scapular position with the subject in standing, **(G)** Morphed adult-sized full-body model **(H)** Detail of the buttock’s region near the ischial tuberosity with a higher mesh density (about 10 mm for mesh size).

Developing an HBM is a time-consuming and complex process. Therefore, we morphed the PIPER Child model (Beillas et al., 2016) into an adult-sized model (Liu et al., 2020). The morphing target was a male aged 40 years old, 1.74 m in stature, and 79.5 kg in weight (see Supplementary Appendix SA1 for more detailed anthropometric dimensions). Different types of data were collected on the target person (Figure 1). The target person’s spine and pelvis geometric models (Figure 1B) in a seated position with a seat pan to backrest angle (SP2BA) of 106° (Figure 1A) were obtained from an MRI study by Beillas et al. (2009). His external body shape was scanned in a similar seating condition as the MRI study with a hand-held laser scanner (Figure 1D) in the present study. For the rest of the bones, we used the full-body skeleton of a male with a similar body size from the PIPER open-source repository (www.piper-project.org, subject ID LTE605), which was segmented from the CT scans in a supine posture (Figure 1C). The shape of the ribcage and the location of the scapulae of the target person were manually palpated in a standing posture (Figure 1F). We assumed the positions of these bones were symmetric, only the right side of the subject was palpated: 11 points along the clavicle, 26 points on the ribcage, 17 points on the scapula and 6 points on the sternum, especially showing the outlines of the rib cage, clavicle and scapula. The full skeleton model from the PIPER subject ID LTE605 was then manually positioned and the bones others than the spine and pelvis were morphed interactively using LS-PREPOST until the shape was close to the palpated points and fitted within the skin envelope. Then, this complete skeleton was used as a target for the child morphing.

Once the full skeleton and skin shape were defined for the target person in a seated posture, we used the batch Kriging option of the PIPER software to facilitate the morphing. When morphing the child model into our target male adult, at first, only the node coordinates were changed and the mesh was kept the same (number of elements, connectivity). For the bones as well as for the regions away from the regions of interest, a same mesh was kept. For the regions of interest for comfort application such as the buttocks, we refined the mesh size of the soft tissue under the sitting bones with an element size of around 10 mm (Figure 1H). Then, the mesh was simplified in regions of limited interest (e.g., internal organs) and made symmetrical. As the internal organs of the abdomen (solid organs) and brain are away from the region of interest for seating, they were removed to save computational time. They were replaced by a constant pressure volume (linear_airbag_fluid) and their mass was distributed onto the envelope. This approach was already used for some parts of the abdomen and is used in other models (e.g., Beillas and Berthet, 2018). To maintain the posture, we constrained the following bones into a single rigid body so that no relative movements were allowed between them: the skull and cervical spine, the calf and foot (left and right), the upper limb bones including the humerus, radius, ulna, hand (right and left bones). Altogether, this reduced the number of deformable elements in the model from about 488,000 to 395,000. As there were different targets for the skin and bones, the soft tissue thickness changed between the child and the adult. For the soft tissue below the IT, as no direct measurement was available from the target subject, a thickness of 40 mm was selected in line with the range from Wang et al. (2021). The adult HBM is shown in Figure 1G.

Regarding the structure of the model, most initial choices in the original child model were kept. Joints for which there is only an interest in terms of kinematic contribution are modelled using 6 degrees of freedom beams (e.g., lumbar and thoracic spine, elbow, knee and ankle). Others are modelled using contacts, ligaments and capsules (e.g., shoulder, hip). These choices are expected to be compatible with the current application, i.e., they allow model positionning and postural change as well as load transfer between skeletal structures during simulation. The simple model used for the spine is also compatible with the choice that the load in the intervertebral discs is not investigated. Most soft tissues are attached to their bone using coincident nodes. This helps with compressive load transfer while keeping the model simple. Exceptions include 1) the scapula which can slide on ribcage 2) tissues near the joints (e.g. shoulder, hip, elbow, knee) which can slide with respect to the skeleton as a direct attachment would stiffen the response and 3) the tissues near the ITs as it was attempted to simulate the muscle sliding observed in some recent MRI studies (Sonenblum et al., 2015; Sonenblum et al., 2018; Wang et al., 2021). Finally, the muscles and adipose tissues were lumped together in a homogeneous isotropic material called flesh as in the original model. This part is mainly loaded in compression. As the model is passive, muscle lines of action were not modelled and the beams initially representing them in the neck were removed. The skin was simulated with a 1 mm thick shell elements with a deformable material.

Concerning material properties, the bones were simplified as rigid bodies and soft tissue properties relevant to the interaction with the seat were also adapted. The soft tissue properties of the child model’s tabular law (MAT_SIMPLIFIED_RUBBER/FOAM) were selected to be stable and adequate for high rate and high stress conditions encountered in crash. The initial child law was found too stiff for the low rate and gravity loading associated with seating comfort. Their properties were changed to a low modulus Neohookean model as in Janák (2020) based on the results from Comley and Fleck (2012). Their material properties are summarized in Table 1.

**TABLE 1:** Material properties.

Materials	Type	Density (kg/mm^3^)	Parameters	
Bones	Rigid (MAT_020)	1.70E-06	Young’s modulus	6 GPa
			Poisson’s ratio	0.3
Soft tissue	Neohookean (MAT_077)	9.00E-7	Poisson’s ratio	0.499
			Shear modulus	Mu1 3E-6 GPa
				Alpha1 2.0
Skin	Elastic (MAT_034)	1.00E-6	Young’s modulus	2.5E-3 GPa
			Poisson’s ratio	0.45
Seat pan foam	low desnity foam (MAT_057)	5.00E-8	Tensile Young’s	1.04E-4 GPa
			HU	0.65
			SHAPE	8
Backrest foam	low desnity foam (MAT_057)	4.90E-8	Tensile Young’s	8.75E-5 GPa
			HU	0.65
			SHAPE	5

To enable soft tissue sliding under the ITs, the method implemented and tested in Beillas and Berthet (2018) for another model was used. First, the soft tissues surrounding the ITs were separated and offset from them with a small arbitrary distance of 0.2 mm. Then, a contact allowing sliding while maintaining the distance between the two components was defined (tiebreak contact with option 4). Frictionless coefficients of friction (COF) between the soft tissues and the ischial bone were used. To study the effect of soft tissue sliding on soft tissue compression, a model without muscle sliding was also developed for comparison.

The mass distribution was aligned with Huang et al. (2015) by adjusting the density of soft tissues and bones. The head has 7.4% of the full body mass. For the upper limbs, the proportion is 4.4% for each side. The trunk and lower limbs make 51.5% and 16.2% of the body mass, respectively.

## 3 Model validation

### 3.1 Experiment with four seat configurations

**FIGURE 2:**
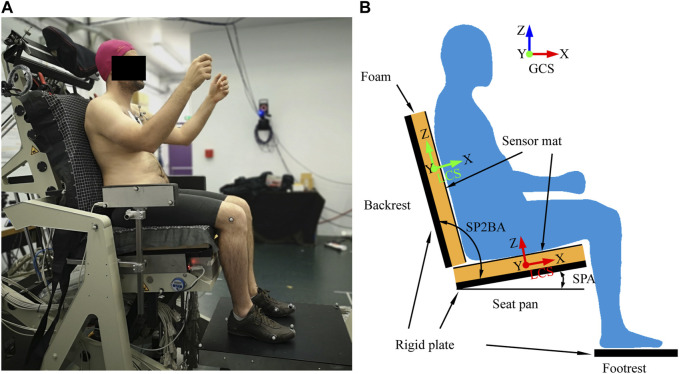
Experimental set-up **(A)** and definition of the global (GCS) and local coordinate systems **(B)**.

To validate the model, an experiment was carried out with a reconfigurable experimental seat (Figure 2). The experimental seat can simulate different seat configurations thanks to 13 motorized adjustments and measure all contact forces (Beurier et al., 2017). The participant was the target male for the model development. Two wooden flat rectangular plates covered by two different 50 mm thick foam cushions were used as seat pan (550 × 550 mm) and backrest (620 × 565 mm). To measure the contact pressures, two pressure mats (XSENSOR, X3 PRO V6, Canada) were attached to the foam with double-sided tape and clips. Four seat configurations (Figure 2) were tested by varying the seat pan angle (SPA) from 0° (horizontal) to 15° with the step increase of 5° while the seat pan to backrest angle (SP2BA) was kept at 100° corresponding to the seating configuration used in the MRI study by Beillas et al. (2009). They were selected to represent the existing seats used in transportation. For each configuration, the seat pan length was adjusted to keep the distance between the front seat edge and the knee hollow at around 40 mm. The participant could adjust the foot support height for comfortable seating.

### 3.2 Foam cushion property test and validation

The MAT_57 (Mat Low Density Foam) material model of LS-DYNA (Livermore Software Technology Corporation in Livermore, CA, United States) was used to simulate the seat and backrest cushions. To use this model, material density, compression curve, tensile Young’s modulus, hysteretic unloading factor (HU), and shape factor for unloading (SHAPE) need to be known. The decay constant for modeling creep in the reloading (BETA) was set to the default value (0.0). The ISO standard (ISO:2439) was applied for the compression test of a 50 × 50 × 50 mm sample. The test was performed on an INSTRON machine with a constant compression velocity of 100 mm/min and stopped at 80% compression with respect to its initial thickness. For the tensile test, the ATSM 3574-17 standard was applied with a 12.5 × 25 × 100 mm specimen at a constant loading speed of 500 mm/min until its failure.

The HU and SHAPE factors were identified with the help of a FE foam model simulating the compression test (Supplementary Appendix SA2). By trial and error, we found that the simulated and experimental compression curves matched well with SHAPE and HU combinations of (8 and 0.65) and (5 and 0.65) for the seat pan and backrest cushions, respectively. The material property parameters of the seat and backrest foams are summarized in Table 1.

After identifying the foam parameters, the seat pan and backrest foam cushion properties were validated by simple compression tests with three different masses before applying them to seating simulations with the adult HBM. It was found that a scaling factor of 1.15 should be applied to the experimental stress-strain curves for both seat pan and backrest cushions (see Supplementary Appendix SA3).

### 3.3 Simulations with the adult HBM

**FIGURE 3:**
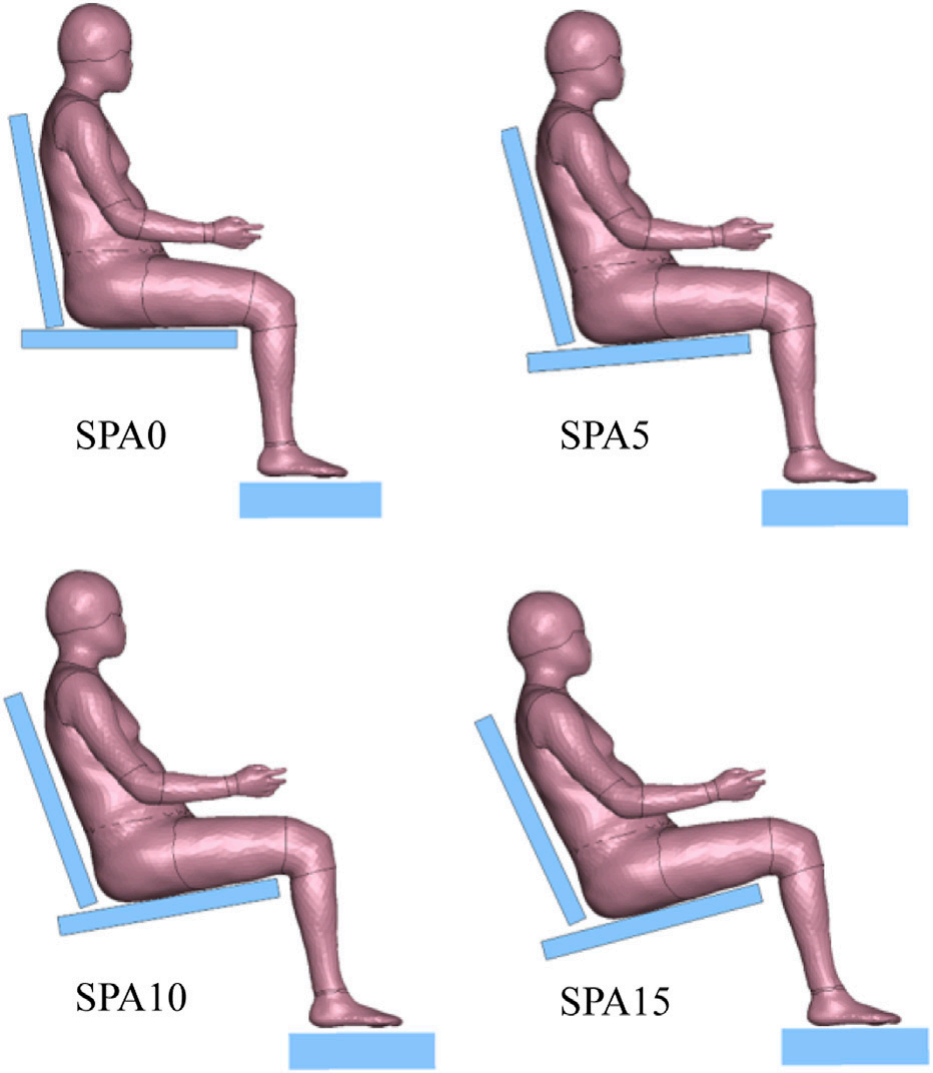
Pre-positioned adult HBM for the four experimental seating conditions (SPA0, SPA5, SPA10, and SPA15).

The four experimental seating conditions were simulated with the developed adult HBM (Figure 3). The LS-DYNA R11 MPP explicit solver was used on 36 cores of a machine equipped with AMD EPYC 7F72 processors. The time step was 2 µs. Relaxation was used to help limit contact force vibrations. The results (pressure, forces, etc.) were gathered for a duration of 500 ms of seating process, which took about 1 h of elapsed time for each simulation.

### 3.4 Model prepositioning and boundary conditions

To facilitate comparisons, the seat pan and backrest cushions were simulated with a 609.6 × 609.6 × 50 mm block, the same as the pressure mats (48 × 48 sensors for each) used in the experiment for comparison purposes. The two cushions were meshed with hexahedron elements of 12.7 × 12.7 × 12.5 mm. The bottom surface of both cushions was fixed, while the footrest was set to rigid and fixed at the position of corresponding experimental conditions.

In the past, different coefficients of friction (COFs) were used between the human body and seat, with values varying from 0.1 to 0.6 (Grujicic et al., 2009; Du et al., 2013; Al-Dirini et al., 2016; Moerman et al., 2016; Yadav et al., 2021). In this study, the surface-to-surface contact was defined for the backrest and seat pan contacts with a COF of 0.1. A COF of 0.4 was used for the footrest and foot contact as suggested by Derler et al. (2008).

For simulating the four seating conditions, a same initial posture prior to loading was used except for a global rotation around the lateral *y*-axis and the ankle joint angle (Figure 3). The HBM was rotated so that its back and thighs were parallel to the seat back and seat pan. The ankles were rotated so that the foot bottoms were parallel to the foot support surface. A gravity loading of 9.81 m/s^2^ was applied.

### 3.5 Comparison parameters

**FIGURE 4:**
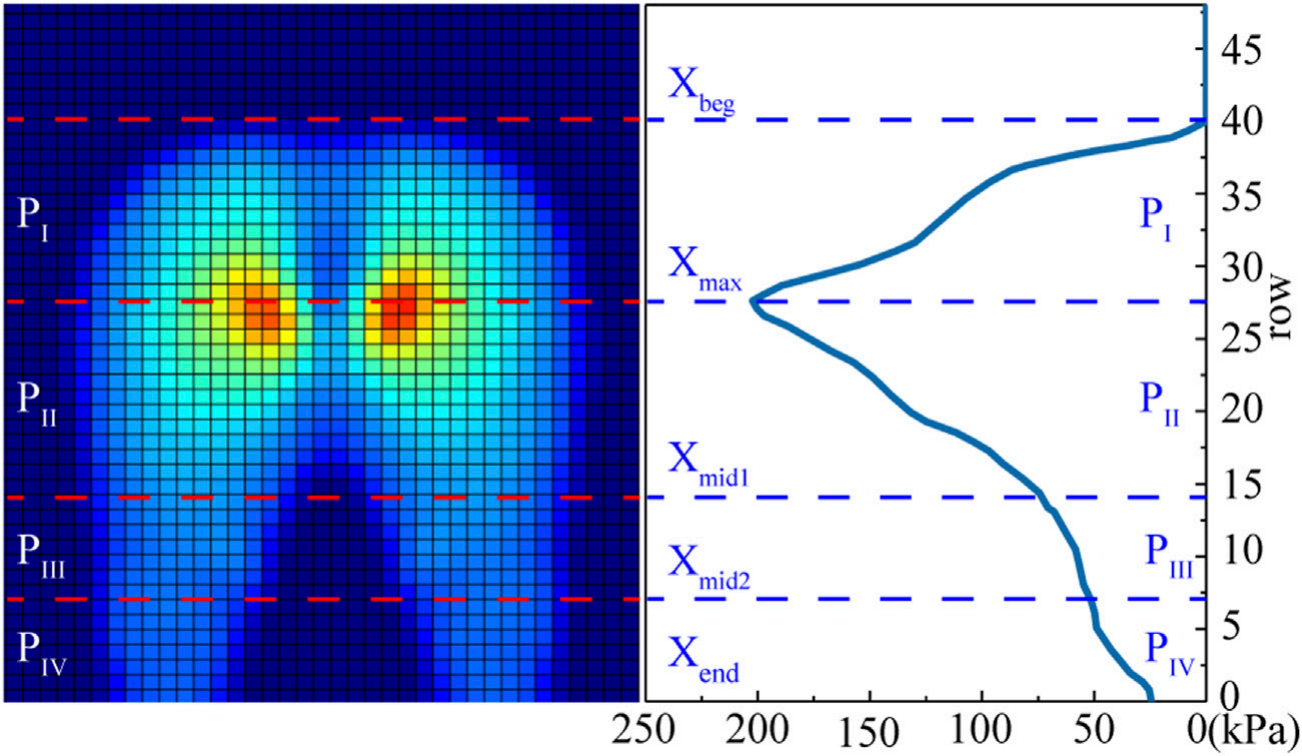
Definition of the four seat pan contact regions (P_I_ to P_IV_) from the lateral pressure profile of pressure distribution. X_beg_ is the first row that the buttocks contacting the sensor mat, X_end_ is the last row that the thighs contacting with the sensor mat X_max_ is corresponding to the row of the peak pressure, X_mid1_ the middle between X_max_ and X_end_, and X_mid2_ the middle between X_mid1_ and X_end_.

The contact forces on the seat pan (Fx_SP, Fz_SP), footrest (Fx_FS, Fz_FS), and backrest (Fx_SB, Fz_SB) were compared between simulations and experiments in both the global (GCS) and local (LCS) coordinate systems (Figure 2B). The sums of all vertical forces including body weight (Sum_Fz) and horizontal forces (Sum_Fx), which should be zero, were verified. In addition to the contact forces, pressure parameters were compared, including contact area (CA), mean pressure (MP), peak pressure (PP), and pressure proportions of four contact areas on the seat pan (P_I_ to P_IV_) (Figure 4). Due to uncertainty in pressure measurements, we applied a correction factor (fcorr) using the trial specific correction method proposed by Zemp et al. (2016). The correction factors for the seat pan and backrest were calculated by comparing the integration of the pressure map over the contact area with the normal force applied on the seat pan or on the backrest measured with load cells. Two pressure profiles were defined in the sagittal plane by summing matrix columns (lateral profile, SOC) and in the frontal plane by summing matrix rows (frontal profile, SOR). Four contact regions on the seat pan were defined from the lateral pressure profile of the seat pan.

**FIGURE 5:**
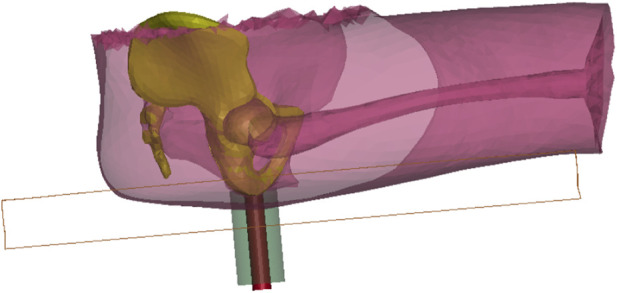
Two regions of interest (ROI) at the ITs defined using two cylinders with a diameter of 20 and 50 mm, illustrated with the unloaded HBM for the SPA5 configuration. The cylinder axes were perpendicular to the seat pan surface and centered at the ischium point closest to the seat. The cylinders were positioned so that the external circle of its upper surface was in contact with the ischium.

To characterize the internal soft tissue deformation, particularly underneath the ITs, two regions of interest (ROI) were defined using two cylinders with a diameter of 20 and 50 mm (Figure 5) as used by Wang et al. (2021). In addition to the mean soft tissue thickness, the tissue volume reduction (R) in ROIs under the IT was computed using the loaded volume (once seated) and the initial volume before loading as follows:

$$R=\left(1-\frac{{Tissue\;Volume}_{loaded}}{{Tissue\;Volume}_{preloading}}\right)\times 100\%$$

## 4 Results

**FIGURE 6:**
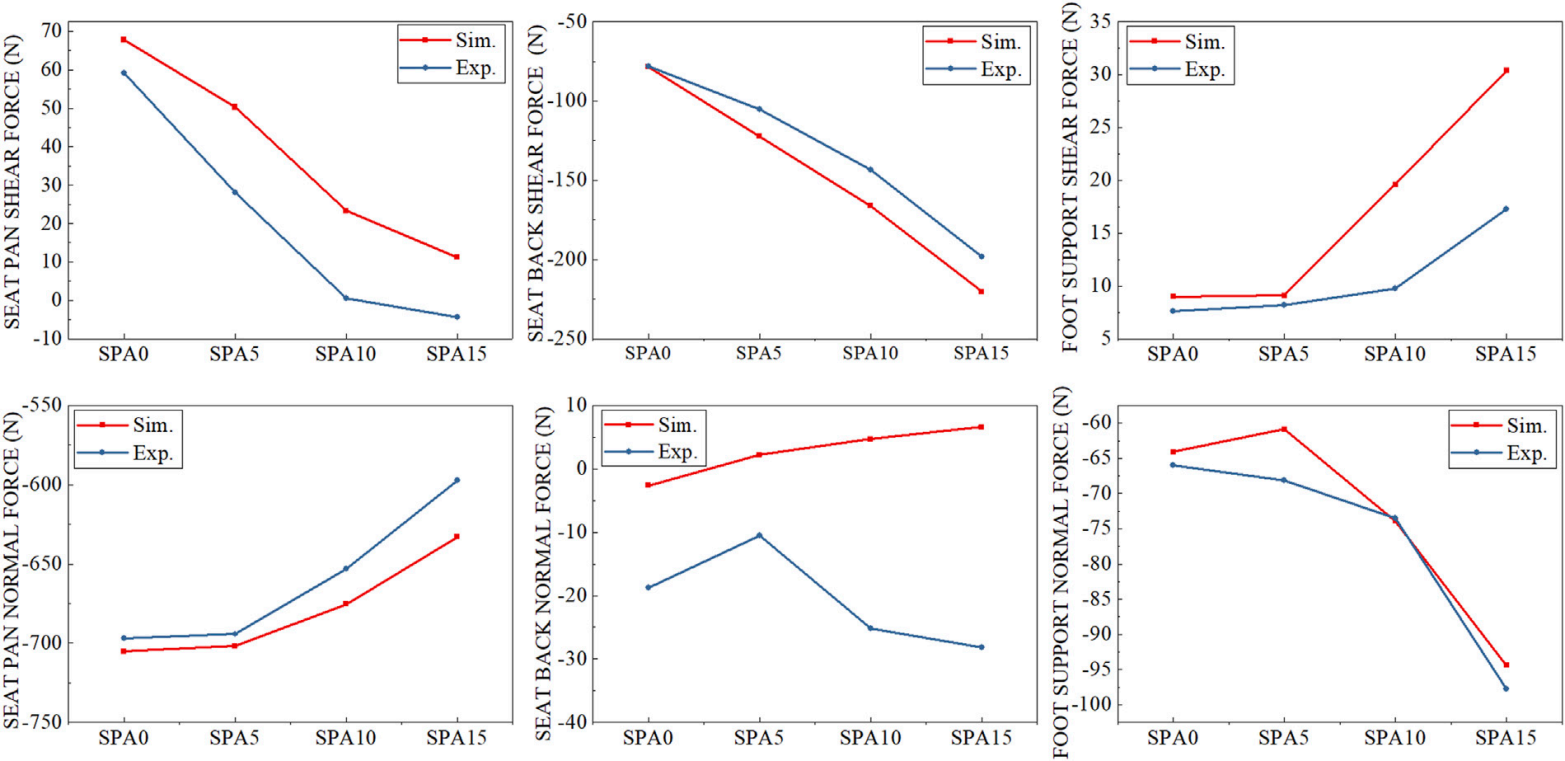
Comparison between simulated and measured shear and normal forces on the seat pan (Fx_SP_L, Fz_SP_L), backrest (Fz_SB_L, Fx_SB_L) and foot support (Fx_FS_G, Fz_FS_G) for the four test conditions (SPA0, SPA5, SPA10, and SPA15).

Overall, simulated contact forces matched well with experimental values and followed same trends when changing seating conditions (Figure 6). Their comparisons are summarized in Table 2. The average differences of the vertical forces were 16.6, −15.5 and 3.0 N respectively on the seat back (Fz_SB_G), seat pan (Fz_SP_G) and foot support (Fz_FS_G), which are very small compared to body weight (784.8 N). Simulated horizontal forces (Fx_SB_G, Fx_SP_G, Fx_FS_G) were also very close to experimental values, but slightly higher in absolute value with an average difference of −22.3, 20.5, and 6.3 N for the seat back, seat pan, and foot support. This also resulted in a slightly higher shear force on the seat pan (Fx_SP_L) with an average difference of 17.3 N. Note that both simulated and experimental values of Sum_Fx and Sum_Fz (including body weight), which should be zero, were less than 10 N.

**TABLE 2:** Comparison between simulated and experimental contact forces (N) as well their differences (D) for the four seat conditions characterized by seat pan angle (SPA). Are shown the forces in the seat symmetry plane applied on the seat back (SB), seat pan (SP) and foot support (FS) in both global (G) and local (L) coordinate systems defined in Figure 2. Sum_Fx, Sum_Fz are the sums of all horizontal forces and vertical forces including body weight.

Seat		Fx_SB_G	Fz_SB_G	Fx_SP_G	Fz_SP_G	Fx_FS_G	Fz_FS_G	Fx_SB_L	Fz_SB_L	Fx_SP_L	Fz_SP_L	Sum_Fx	Sum_Fz
SPA0	Exp	−73.5	−32.0	59.2	−697.0	7.6	−66.0	−78.0	−18.7	59.2	−697.0	6.7	−10.2
	Sim	−76.8	−16.2	67.8	−705.2	9.0	−64.1	−78.5	−2.6	67.8	−705.2	−0.1	−0.7
	D	−3.3	15.8	8.6	−8.1	1.3	1.9	−0.5	16.1	8.6	−8.1	−6.8	9.5
SPA5	Exp	−98.8	−37.3	88.4	−689.2	8.2	−68.1	−105.1	−10.5	28.0	−694.3	2.2	−9.9
	Sim	−118.7	−29.6	111.3	−694.8	9.1	−60.9	−122.3	2.2	50.3	−701.9	1.8	−0.5
	D	−19.8	7.7	22.9	−5.6	0.9	7.2	−17.2	12.6	22.3	−7.6	−0.4	9.4
SPA10	Exp	−126.2	−72.8	113.9	−643.0	9.8	−73.5	−143.5	−25.2	0.5	−653.0	−2.5	−4.3
	Sim	−157.7	−52.4	140.3	−661.1	19.6	−73.9	−166.1	4.7	23.3	−675.4	2.1	−2.6
	D	−31.5	20.4	26.4	−18.1	9.8	−0.4	−22.6	30.0	22.8	−22.4	4.6	1.7
SPA15	Exp	−167.8	−109.4	150.5	−578.2	17.3	−97.7	−198.3	−28.2	−4.3	−597.4	0.0	−0.5
	Sim	−202.4	−87.1	174.7	−608.4	30.4	−94.4	−220.3	6.6	11.2	−632.9	2.6	−5.1
	D	−34.6	22.3	24.2	−30.2	13.1	3.3	−22.0	34.9	15.5	−35.5	2.6	−4.6
All	Exp	−116.6	−62.9	103.0	−651.9	10.7	−76.3	−131.2	−20.7	20.8	−660.4	1.6	−6.2
	Sim	−138.9	−46.3	123.5	−667.4	17.0	−73.3	−146.8	2.7	38.2	−678.8	1.6	−2.2
	D	−22.3	16.6	20.5	−15.5	6.3	3.0	−15.6	23.4	17.3	−18.4	0.0	4.0

For the pressure parameters (Table 3), the simulated pressure proportions in the four regions (P_I_ to P_IV_) defined in Figure 4 had a difference of 5.2%, −1.4%, −13.9%, and −3.1% with respect to the experimental ones. The differences in terms of contact area (CA), peak pressure (PP), and mean pressure (MP) were respectively 5.8%, −6.4%, and 1.2% for the seat pan on average. For the backrest, relative larger errors were observed due to the small magnitude of the pressures. The relative difference (D%) reached 20.2% (12258 mm^2^), 62.1% (2.1 kPa), 3.4% (0.1 kPa) compared to experimental values for CA, PP, and MP on average.

**TABLE 3:** Comparison between simulated and experimental pressure-related parameters for the four seat conditions characterized by seat pan angle (SPA), including contact area (CA), peak pressure (PP) and mean pressure (MP) on the seat pan (SP) and back (SB). P_I_ to P_IV_ are the pressure proportions in the four regions of seat pan pressure defined in Figure 4. Fcorr_SB and fcorr_SP are the correction factors of the measured pressures on the seat back and seat pan.

Seat		CA_SB (mm^2^)	PP_ SB (kPa)	MP_ SB (kPa)	CA_ SP (mm^2^)	PP_ SP (kPa)	MP_ SP (kPa)	P_I_ (%)	P_II_ (%)	P_III_ (%)	P_IV_ (%)	fcorr_SB	fcorr_SP
SPA0	Exp	40,968	3.2	1.9	168,225	13.4	4.1	36.7	42.4	13.4	7.5	1.76	1.38
	Sim	46,935	4.9	1.9	176,774	13.1	4.2	39.2	42.7	11.3	6.7		
	D	5,968	1.8	0.0	8,548	−0.2	0.0	2.5	0.3	−2.1	−0.7		
	D%	14.6	55.6	1.6	5.1	−1.7	0.6	6.5	0.8	−18.8	−10.9		
SPA5	Exp	49,355	3.5	2.1	165,484	14.1	4.2	40.4	40.9	12.1	6.5	1.69	1.48
	Sim	65,968	5.4	2.1	174,354	13.2	4.2	39.1	42.4	11.5	6.9		
	D	16,613	1.9	0.0	8,871	−0.9	0.0	−1.3	1.5	−0.6	0.4		
	D%	34	54.4	−1.0	5	−6.2	0.0	−3.2	3.4	−5.3	6.0		
SPA10	Exp	71,451	3.3	2.0	165,967	14.7	3.9	37.2	43.1	13.8	6.0	1.81	1.54
	Sim	78,387	5.8	2.3	176,451	12.2	4.0	42.2	39.8	11.4	6.7		
	D	6,935	2.5	0.3	10,484	−2.5	0.0	5.0	−3.3	−2.4	0.7		
	D%	9.7	76.2	15.6	6.3	−17.3	0.5	11.9	−8.3	−21.2	10.3		
SPA15	Exp	80,806	3.8	2.5	158,871	12.1	3.8	40.9	42.5	11.5	5.1	1.67	1.64
	Sim	100,322	6.2	2.4	168,871	12.2	3.9	43.5	41.8	10.4	4.3		
	D	19,516	2.4	0.0	10,000	0.2	0.1	2.5	−0.7	−1.1	−0.8		
	D%	24.2	62.5	−1.3	6.3	1.4	3.9	5.8	−1.6	−10.5	−17.7		
All	Exp	60,645	3.4	2.1	164,637	13.6	4.0	38.8	42.2	12.7	6.3	1.73	1.51
	Sim	72,903	5.6	2.2	174,113	12.7	4.1	41.0	41.7	11.1	6.2		
	D	12,258	2.1	0.1	9,476	−0.9	0.0	2.2	−0.5	−1.6	−0.1		
	D%	20.2	62.1	3.4	5.8	−6.4	1.2	5.2	−1.4	−13.9	−3.1		

Soft tissue deformations under the ITs were also calculated for the four seating conditions. The soft tissue thicknesses simulated with and without considering soft tissue sliding are compared in Table 4. Allowing soft tissue sliding reduced the soft tissue thickness under the IT from 17.7 to 15.4 mm and from 23 to 20.5 for the 20 and 50 mm ROIs, with corresponding soft tissue volume reductions R increasing from 49.5% to 57.6% and from 40.3% to 47.9%.

**TABLE 4:** Comparison of simulated soft tissue thicknesses (mm) under the ischial tuberosity before (T_preload) and after loading (T_loaded) for the four seat conditions. The simulations with (Y) and without (N) considering tissue sliding are compared. The average tissue thicknesses were calculated in two ROIs defined in Figure 5.

			ROI 20 mm			ROI 50 mm	
Seat	Tissue Sliding	T_ preload	T_ loaded	R	T_ preload	T_ loaded	R
SPA0	Y	38.8	14.1	60.8	41.0	19.3	50.8
	N	38.9	16.8	51.8	41.2	22.1	42.4
SPA5	Y	38.8	15.4	58.5	41.1	20.5	49.1
	N	38.8	17.7	51.5	41.0	23.1	42.4
SPA10	Y	38.8	16.0	56.1	41.1	21.1	46.3
	N	38.9	18.2	47.3	41.2	23.4	37.8
SPA15	Y	38.8	16.0	54.8	41.1	21.0	45.3
	N	38.9	18.0	47.5	41.2	23.3	38.5
All	Y	38.8	15.4	57.6	41.0	20.5	47.9
	N	38.9	17.7	49.5	41.2	23.0	40.3

## 5 Discussion

In this paper, we compared the contact forces and pressure-related parameters simulated using the adult HBM with those obtained experimentally from the person whose data was used for the model development. Four seat configurations, with the seat pan angle varying from 0° to 15° and seat-to-back angle fixed at 100°, were tested.

### 5.1 Contact forces

Results show that the adult HBM could correctly simulate the contact forces on the backrest, seat pan, and foot support with an average error of less than 22.3N and 15.5 N in the horizontal and vertical directions, which is small considering the body weight (784.8 N). However, simulated horizontal forces on the three contact surfaces were systematically higher than experimental results, leading to a systematically higher shear force on the seat pan (Fx_SP_L). Wang et al. (2018) investigated the relationship between seat contact forces and perceived discomfort. They found that a seat configuration with a self-selected seat pan angle reduced shear force on the seat pan. For the seat configuration SPA0 tested in the present work, the seat back angle was 10° and the seat pan was horizontal. For a seat back angle of 10°, the preferred seat pan angle was about 5.9° on average according to Wang et al. (2018). Compared to a horizontal seat pan, the shear force on the seat pan (Fx_SP_L) could be reduced from 8.56% to 4.61% of body weight, a reduction of about 4% of body weight, representing a shear force of about 32 N on the seat pan surface for the target person in the present study. Due to the relatively small shear force compared to the normal one, especially on the seat pan surface, a small difference in shear may be of importance for discomfort perception.

Higher simulated horizontal forces may be due to an inappropriate choice of boundary conditions including loading, initial model positioning, coefficients of friction of different contact surfaces as well as due to the effects of passive models. Sitting was simulated by releasing the HBM, which was pre-positioned slightly above the seat without considering muscle activation in postural control. However, this simulation did not accurately represent how a person would sit on a seat. When a person sits into a seat, they will put their buttocks on the seat first, using their hands pressing the armrests or other fixed objects (e.g., steering wheel for car seat) to guide the body movement, and then move their back to the backrest. One or two small repositioning motions are often necessary if they are not comfortably seated initially. Repositioning not only re-adjusts the posture, but also may reduce the shear forces between body and seat. However, small repositioning is challenging to simulate by just releasing the body. This was not addressed in previous simulation studies either. Therefore, in the future, a more realistic seating process should be explored for HBM simulations.

The body seat interface in the present study was complex as the foam cushion was covered with an XSESNOR pad. The participants only wore shorts without any cloth on the upper body, which may have resulted in a COF difference between the seat pan and backrest contact surfaces. Possible effects of COFs on contact forces also need to be studied.

### 5.2 Pressure parameters

In terms of contact area, peak, and mean pressure, the simulation matched well with the experiment for the seat pan, i.e., the magnitudes of their differences were limited. The global contact area was always higher by simulation (5.8% on average, Table 3). The simulated contact areas under the thighs were however smaller than the experiment values if we compare the simulated and experimental pressure distributions (Supplementary Appendix SA4). One reason could be that the material properties of soft tissues might not be appropriate. According to Scott et al. (2020), the apparent soft tissue material properties could be significantly different even for the same subject from different positions. The soft tissue of the adult HBM was simulated with an isotropic homogeneous Neohookean material not accounting for regional difference of properties, the presence of muscles, their fascia, or the possible sliding between structures. Another reason might be related to the initial state of soft tissues. Both soft tissue thickness and initial strain states in a sitting posture prior to loading are not known and there is limited data available in the literature. For the region under the ITs, the soft tissue thicknesses in the ROI of 20 mm and 50 mm were adjusted to 39 and 41 mm based on the observations from four males using an open MRI by Wang et al. (2021). The muscle tension, which is expected to be limited while sitting, is not known either.

As far as the pressure parameters of the seat back are concerned, much larger differences in contact area (SB_CA) and shape between simulation and experiment were observed compared to seat pan pressure parameters (Table 3; Supplementary Appendix SA4). The lower back was in contact with the seat in the simulation while this was not observed in the experiment in any of the four seat configurations. One reason could be that the HBM was positioned too backward on the seat pan, and that the actual subject’s trunk-to-thigh angle was larger than the 100° used to pre-position the model. This makes the back less parallel to the seatback surface, resulting in a single-location contact in the upper back region. A larger contact area on the seat back may be one of the reasons for a lower backrest mean pressure (MP_SB). The simulated backrest peak pressure was 2.1 kPa larger than the experiment on average. The peak pressure occurred in the lower scapula area with the presence of sharp edges and very thin soft tissues. As explained in the model development, there was no ribcage and scapula in the MRI geometry information of the target subject. Target information was obtained by palpating the subject in a standing posture (Figure 1F). This could lead to errors in the shape and position of the ribcage and scapulae in the seated posture. In addition, muscle activity, which was not simulated, may play a more important role in human backrest interaction than in the human seat pan interaction due to the need to maintain the posture.

The pressure mapping system used in the present study had an accuracy of ±10% of scale for a range of 1.4–27 kPa according to the product description by the manufacturer. Similar to the correction method used by Zemp et al. (2016), we applied a correction factor to match measured normal forces on the seat pan and backrest. Because of this, comparing the simulated and measured pressure values needs to be done with care. We think it is more important that simulated pressures follow the same trends when changing seating conditions, which is the case for the present study.

### 5.3 Soft tissue deformation

Soft tissue deformation (especially those below the ITs) is considered to be one of the major contributing factors to discomfort and injury (Ceelen et al., 2008). Using an upright MRI system, some researchers recently observed that the gluteus muscle was displaced from the IT once seated (Sonenblum et al., 2013; Sonenblum et al., 2015; Sonenblum et al., 2018; Wang et al., 2021). However, to our knowledge, none of the existing models for comfort considered soft tissue sliding. In the study by Wang et al. (2021), the FOAM condition, corresponding to a seat configuration with SPA of 7° and SP2BA of 105° using a foam of 50 mm on the seat pan, was close to the condition SPA5 in the present study. They observed that four male participants had a tissue volume reduction R varied from 54.8% to 69.6% for 50 mm ROI and from 60.2% to 74.7% for 20 mm ROI. For SPA5, simulated R values were 49.1% and 58.5% for ROIs of 50 mm and 20 mm using the model with soft tissue sliding. Without considering soft tissue sliding, we obtained smaller soft tissue compressions, which were 42.4%, and 51.5% for ROIs of 50 mm and 20 mm. Compared to the MRI observations from four subjects (Wang et al., 2021), the simulation showed the same variation trend when changing seating condition, but lower soft tissue compression ratio. The value R with sliding in ROI 20 mm was 57.6% for an experimental range between 60.2% and 74.7% observed from four subjects. Therefore, considering the large subject-to-subject variability, the simulation value can be considered close to the experimental range although on the lower side. The reasons for the subject-to-subject variability (despite similar initial thickness for some of them) are unknown but would be helpful to understand the modelling requirements. In the meantime, a sensitivity study on the initial soft tissue thickness, initial strain in the tissues due to gravity and material properties could certainly be of interest in the future. While the simulation of the sliding may not be perfect in the current implementation, the tissue sliding may therefore help simulate the tissue response in the ischial region and, from a modelling standpoint, help prevent the use of excessively soft material properties in the region to match the external compression.

### 5.4 Human body modeling

The level of anatomical details to be modeled depends on application. The open source approach enables future changes and evolutions in the structures using the model as a basis including by other researchers. The current modeling choices have to be considered as starting choices reflecting the first intended applications in transportation. This includes the simulation of the interaction with the seat pan and the seat back including reclined seating configuration. Partial buttock thigh models developed by Verver et al. (2004), Yadav et al. (2021) are unable to simulate the interaction with the seatback. Therefore, it is important to include the trunk and spine in the model. However, as we are not necessarily focusing on the internal loads in the spine, the modelling was simplified using either articulated vertebrae or rigid segments. This can be changed if needed.

Concerning the muscles, it was hypothesized that for relaxed, static seating (including reclined), the muscular activity would play a limited role, and that differences of muscular activities would be small between seating configurations. Hence, the muscles activity was not modelled and active muscles that were present in the neck of the original model were removed. This assumption could evolve depending on application (e.g., dynamic comfort, etc.).

The passive contribution of the muscles and other soft tissues was modelled but it was assumed that small internal differences in structures (e.g., separating fiber bundles, fascia) would not affect the forces (including shear), or pressure as long as the overall stiffness of the combined structure was modelled. The possible sliding of the structure was considered near the ischium based on recent observations by Wang et al. (2021) and others.

**FIGURE 7:**
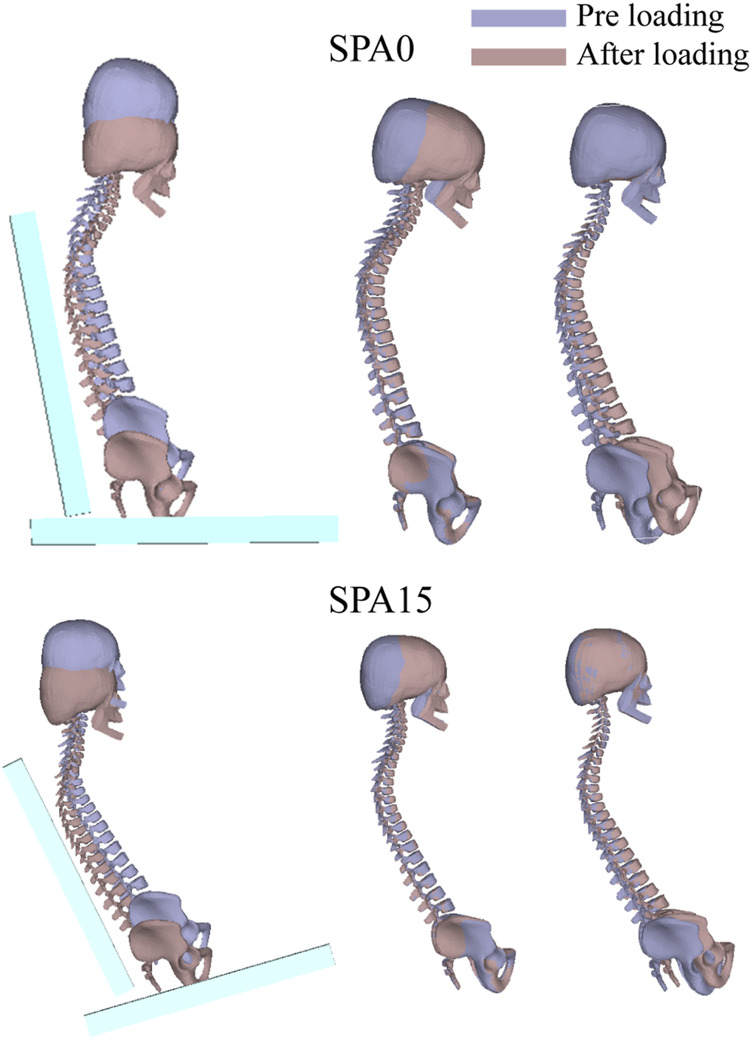
Comparison of simulated spine curvatures before and after loading for SPA0 and SPA15 in the seat coordinate, after alignment with respect to the skull and the pelvis.

In the present study, a same initial posture prior to loading was used to simulate the four seating conditions. As explained in Section 2.1, the initial curvature of spine prior to loading was derived from the target subject’s MRI data in a seating posture from our previous study (Beillas et al., 2009). In that study, the seat pan to seat back angle was 106°, which is slightly more than in the current study (100° for all conditions). However, the seat was only covered with a very thin foam while a 50 mm foam was used in the current study. The two seated postures are therefore expected to be similar, and no adjustment was made prior to the simulations. Only a global rotation around the lateral *y*-axis was applied so that the model’s back and thighs were parallel to the seat back and seat pan surfaces for the four seat configurations. It is interesting to see that the loading by gravity resulted in a rearward pelvis tilt and a slight change of curvature in the lumbar and thoracic regions after loading (Figure 7). Recall that the cervical vertebrae and skull were constrained together as a rigid body and only the rest of spinal joints were allowed to rotate during loading.

### 5.5 Implication in developing a seating (dis)comfort assessment tool

Since the proposition of the conceptual model of sitting comfort and discomfort by Zhang et al. (1996), it is well accepted that comfort and discomfort are two distinct concepts. Discomfort is associated with biomechanical factors such as posture, soft tissue deformation, and pressure distribution, while comfort is associated with feelings of relaxation and wellbeing. We, as many researchers, believe that discomfort could be explained by some objective parameters including pressure and other biomechanical parameters. Zemp et al. (2015) made an extensive review on the question whether pressure measurements are effective in the assessment of office chair comfort/discomfort. Though the question cannot be definitively answered due to the limited availability of data, they suggest that the peak pressure of the seat pan, the pressure distribution of the backrest and the pressure pattern changes (seat pan and backrest) appear to be reliable for quantifying comfort or discomfort. The adult model developed in the present study is just a first step, further developments and more research is needed to achieve a virtual seating comfort assessment tool. To better represent a target sitter population, models of various sizes have to be generated. The present adult model could be used as a reference using a morphing tool as proposed in PIPER. More importantly, relevant biomechanical evaluation criteria are still missing. This requires investigations, which establish the relationship between subjective feeling and objective biomechanical parameters.

### 5.5 Limitations

First, as a first validation study, only four seat configurations were tested with the seat pan angle varying from 0° to 15° while the seat pan to backrest angle was fixed to 100°. Therefore, in the present study, the same spinal curvature including the pelvis was used to define the model’s initial sitting posture. More reclined seating conditions for relaxing postures need to be validated.

Secondly, boundary conditions including model’s pre-positioning, loading process, and COFs, need to be further investigated. Measuring the seated body position by palpating anatomical landmarks could also be helpful in defining a more realistic model initial position for simulation.

Thirdly, as already mentioned, the model’s trunk including the scapulae and ribcage may need to be further improved. An appropriate trunk model will be important for the seats with an increased ability to recline, as more body weight will be supported by the seat back. In the present study, we focused more on the model’s adaptation to study the loading of the body by the seat pan. As the developed adult HBM will be open-sourced and accessible to other researchers, further model improvement will be facilitated.

Fourthly, the seat pan and backrest cushions were tested under well-controlled compressions by three masses for validating the identified foam properties (Supplementary Appendix SA3). Simulated compressions were higher than experimental values for two higher masses. A scaling factor of 1.15 was therefore applied to the initial foam stress-strain curves for a better match. The exact source of this discrepancy is unknown. Further investigation is needed to understand its causes and to characterize foam properties. Nevertheless, we verified that a 15% difference on the foam properties had almost no effect on soft tissue compression at the IT area and very limited effect on contact forces and pressure parameters. The maximal difference in contact forces was less than 7 N, while the differences in peak pressure were −0.9 kPa (−6.4%) and 2.1 kPa (62.1%) for the seat pan and backrest cushions.

Lastly, the effect of soft tissue material properties is not investigated. A sensitivity study is needed to understand how these parameters affect the responses to the loading when seated. These effects should also be put in the perspective of variations expected for a large population of seat users.

## 6 Conclusion

Developing an adult HBM is time-consuming and collecting all the required data from a same target person in a seated position is difficult. In this work, we developed a mid-sized male adult full-body model by morphing the PIPER Child model with target information from different sources. To the best of our knowledge, this is the first validation study of an adult HBM against contact forces and pressure parameters in several seat configurations used in offices and transports. We also introduced soft tissue sliding under the ITs for the first time in a model, which resulted in a higher soft tissue compression in agreement with recent observations using an upright MRI by several researchers. To facilitate its reuse and improvement, the developed adult HBM, called PIPER adult comfort (version 1.0), will be released at the time of publication under the same open-source license as the PIPER Child model (GPL v3 license with open science and liability clauses) at the PIPER repository (http://piper-project.org/).

## Data Availability

The original contributions presented in the study are included in the article/Supplementary Material, further inquiries can be directed to the corresponding author.
