# Supplementary material for: PIPER adult comfort: an open-source full body human body model for seating comfort assessment and its validation under static loading conditions
Source: Front Bioeng Biotechnol. 2023 May 30;11:1170768. doi: 10.3389/fbioe.2023.1170768 (PMC10267746; doi:10.3389/fbioe.2023.1170768)
Supplement: Supplementary file 1 [file DataSheet1.pdf]

1 Appendix

1.1 A1 Anthropometric dimensions of the target male adult used for model development

Table A 1. Anthropometric dimensions of the target male adult

Measure	Unite	Posture	Value
Stature	mm	Standing	1738
Weight	kg	Standing	79.5
BMI	kg/m^2	-	26.3
Head-to-floor height	mm	Seated	1340
Eye-ground height	mm	Seated	1210
Shoulder-ground height	mm	Seated	1050
Elbow-ground height	mm	Seated	680
Seat height	mm	Seated	410
Popliteal height	mm	Seated	430
Buttock-popliteal length	mm	Seated	520
Buttock-knee length	mm	Seated	645
Forearm-fingertip length	mm	Seated	460
Hip breadth	mm	Seated	403
Waist breadth	mm	Seated	300
Elbow-to-elbow breadth	mm	Seated	528
Thorax breadth	mm	Seated	368
Shoulder (bideltoid) breadth	mm	Seated	447
Stature (with shoes)	mm	Standing	1754
Weight (with shoes)	kg	Standing	80

1.2 A2 Results of foam property tests and foam mechanical property identification

Figure A1 shows the experimental setup for foam compression and tensile tests as well as the corresponding strain-stress curves. To identify the HU and SHAPE factors of the material law MAT_57, a FE foam model (Figure A2 (A)) with the same dimension as the test specimen (50 x 50 x 50 mm) was developed to simulate the compression test. By testing three SHAPE values of 5, 8, and 10 (values recommended in the LY-DYNA user’s manual are from 5 to 10) with HU fixed at 0.6, we found that SHAPE had little influence on the loading-unloading curve (Figure A2 (B)). Then simulations were made by varying HU from 0.1 to 0.8 with SHAPE primarily fixed at 5. By trial and error, we found that the simulated compression curves matched pretty well with the experimental ones with a SHAPE and HU combination of (8 and 0.65) and (5 and 0.65) for the seat pan (Figure A2 (C)) and backrest cushions (Figure A2 (D)). The differences in the area covered by the loading curves were 2.85% and 1.83% for backrest and seat pan cushions respectively, while for unloading phase they were 13.06% and 7.53%.

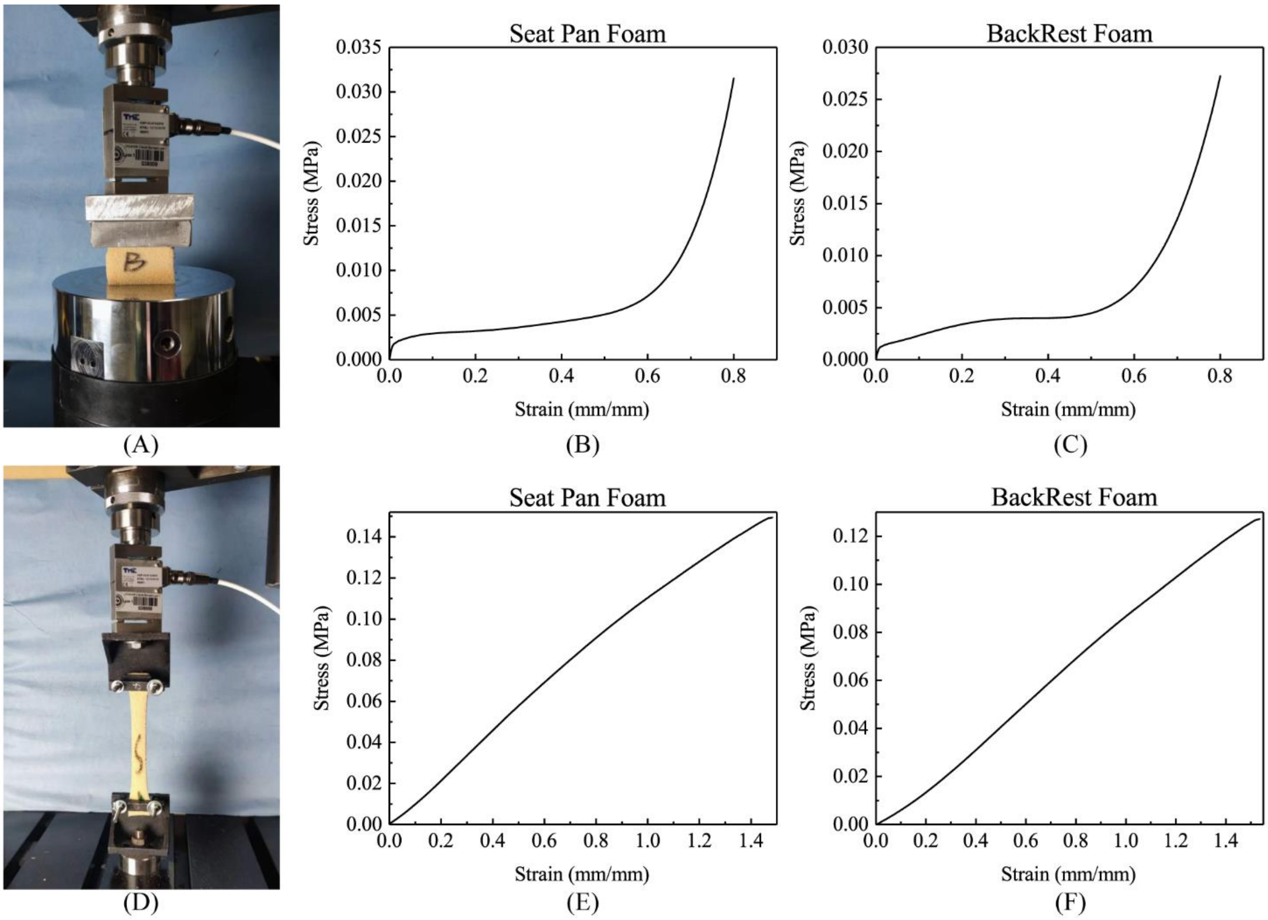

Figure A1. Foam compression test set-up (A) and corresponding engineering strain-engineering stress curves for the foams used for the seat pan (B) and the backrest (C), as well as the tensile test set-up (D) and the corresponding results for the seat pan (E) and the backrest (F) foams

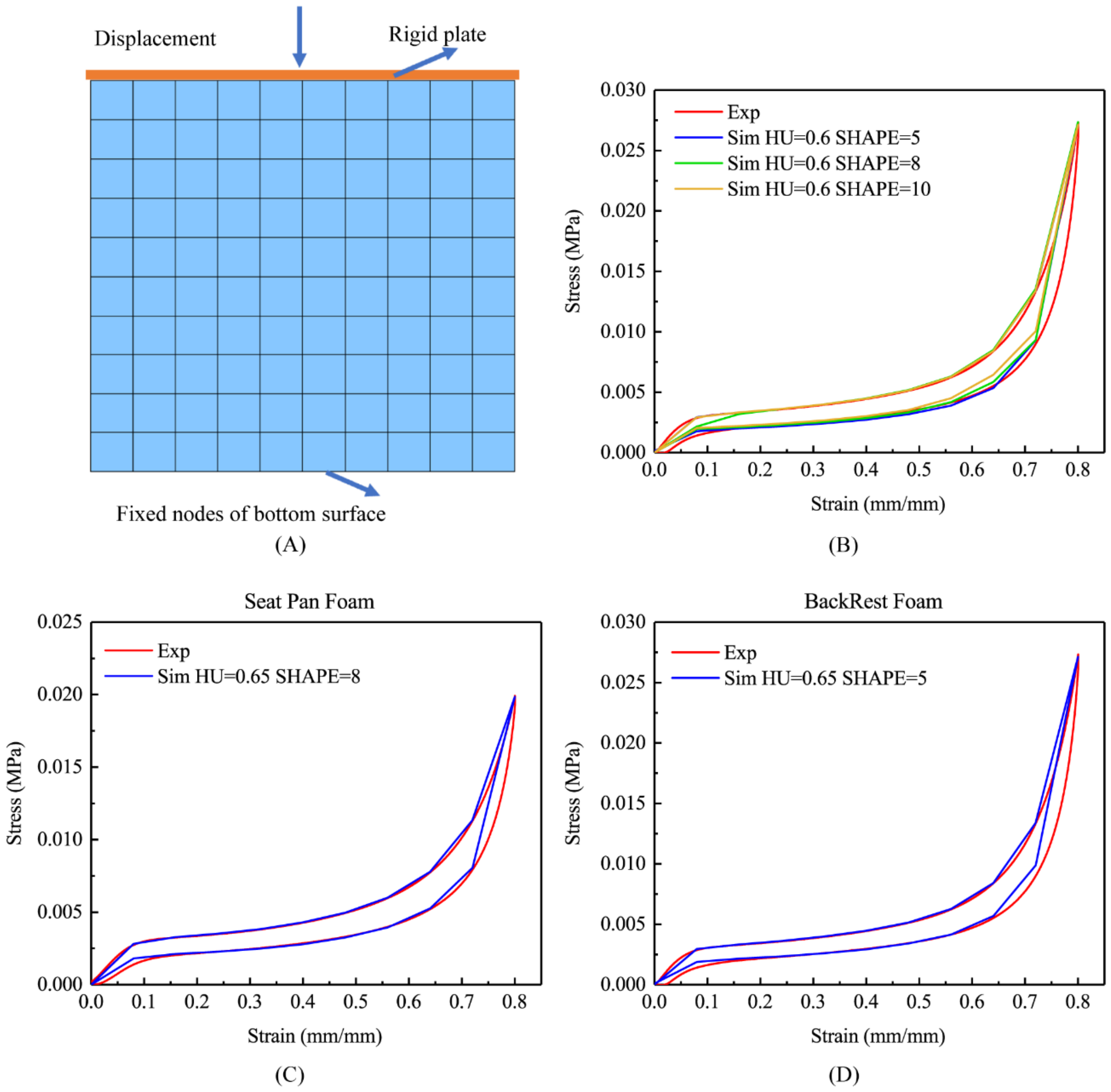

Figure A2. HU and SHAPE factors identification: (A) foam specimen FE model, (B) Experimental and three simulated loading-unloading strain-stress curves, (C) and (D) are experimental and simulated loading-unloading curves with the identified values of HU and SHAPE for the seat pan and backrest foams

1.3 A3 Validation of foam properties by simple compression tests with three different masses

To verify if the identified foam properties can be used to simulate the response of the seat and backrest cushions to a well-controlled compression loading, the cushions were placed on a horizontal wooden support and compressed with three different masses of (25.53, 35.75, 45.75 kg). They were placed on a wooden board with a weight of 0.8 kg as shown in Figure A3(A). A laser light and an inclinometer helped to adjust the mass position to ensure an even loading. The foam compression was estimated by the position of the markers attached to the wooden board measured by a VICON motion capture system (Oxford, UK). Each mass condition was tested three times. For FE simulation (Figure A3(B)), the nodes of the bottom surface of the foam block were fixed and the wooden board was set to rigid. An automatic surface-to-surface contact was defined for the board and foam interface. As summarized in Table A2, the simulated compressions were generally larger than the experimental ones. The

exact source of this discrepancy is unknown. Possible reasons include differences in boundary conditions (e.g. friction coefficient) or inhomogeneities of the foam specimen as they were cut from the edge of the cushion foam. A scaling factor of 1.15 was found by trial and error and applied to the experimental stress-strain curves for both seat pan and backrest cushions. As the loading applied by the adult HBM is more similar to the experiment with the plate than with the cubic sample, the 1.15 corrective factor was also used for further simulations with the HBM.

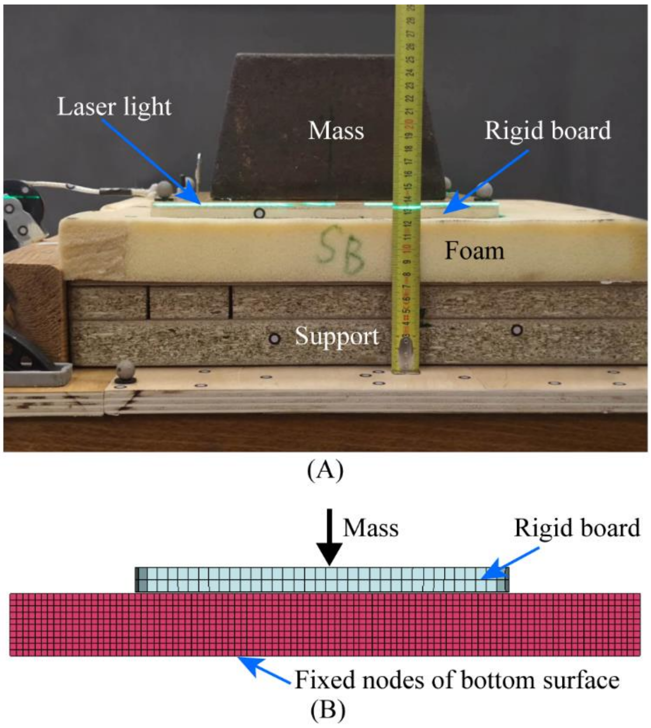

Figure A3. Validation of foam properties: (A) Experiment set up, (B) Corresponding FE models and boundary conditions

Table A2. Comparison of experimental and simulated compressions (mm) of the seat pan and backrest cushions for the compression tests with three masses as well as simulation results after scaling of the experimental compression stress-strain curve.

Foam	Mass (kg)	Exp (mm)	Sim (mm)	Sim after scaling (mm)
	26.33	10	10	9.5
Seat Pan	36.55	17.7	20	18
	46.55	24	27	24
	26.33	9.5	9.5	9
Backrest	36.55	15.6	19	14
	46.55	22.8	26	22.5

1.4 A4 Comparison of simulated and experimental pressure distribution and pressure profiles

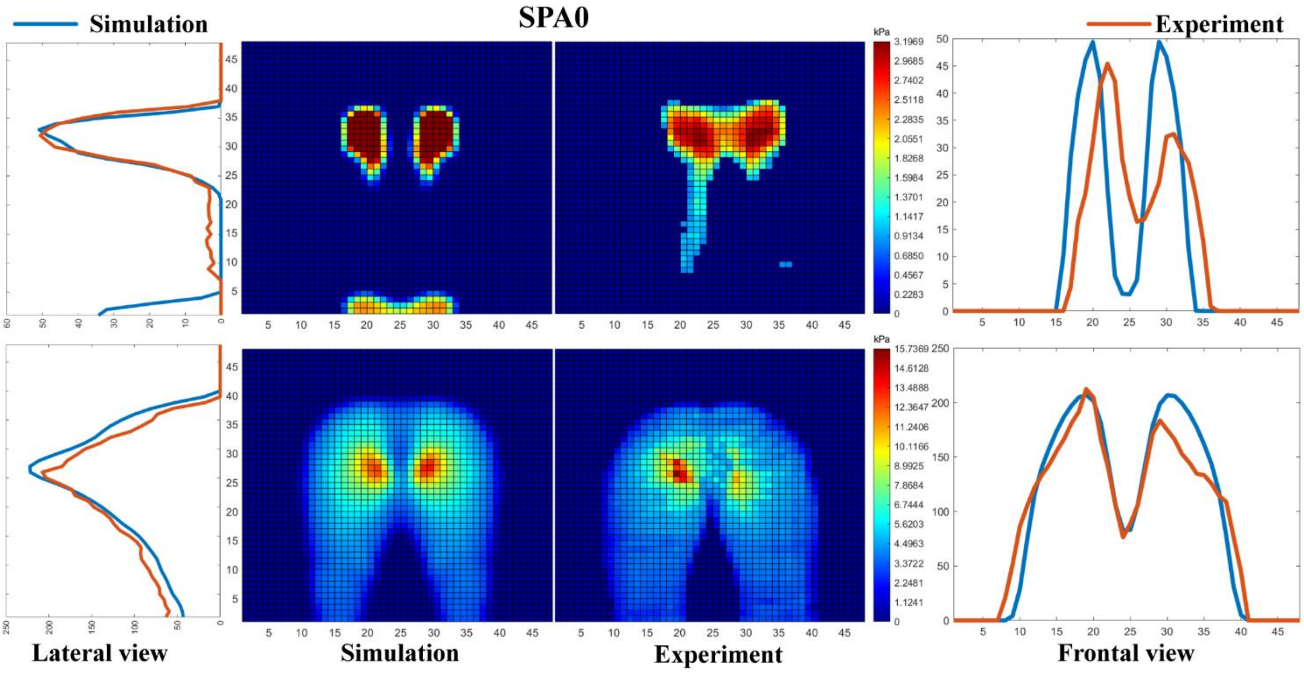

Simulation
SPA0
Experiment
kPa
Lateral view
Simulation
Experiment
Frontal view

(A)

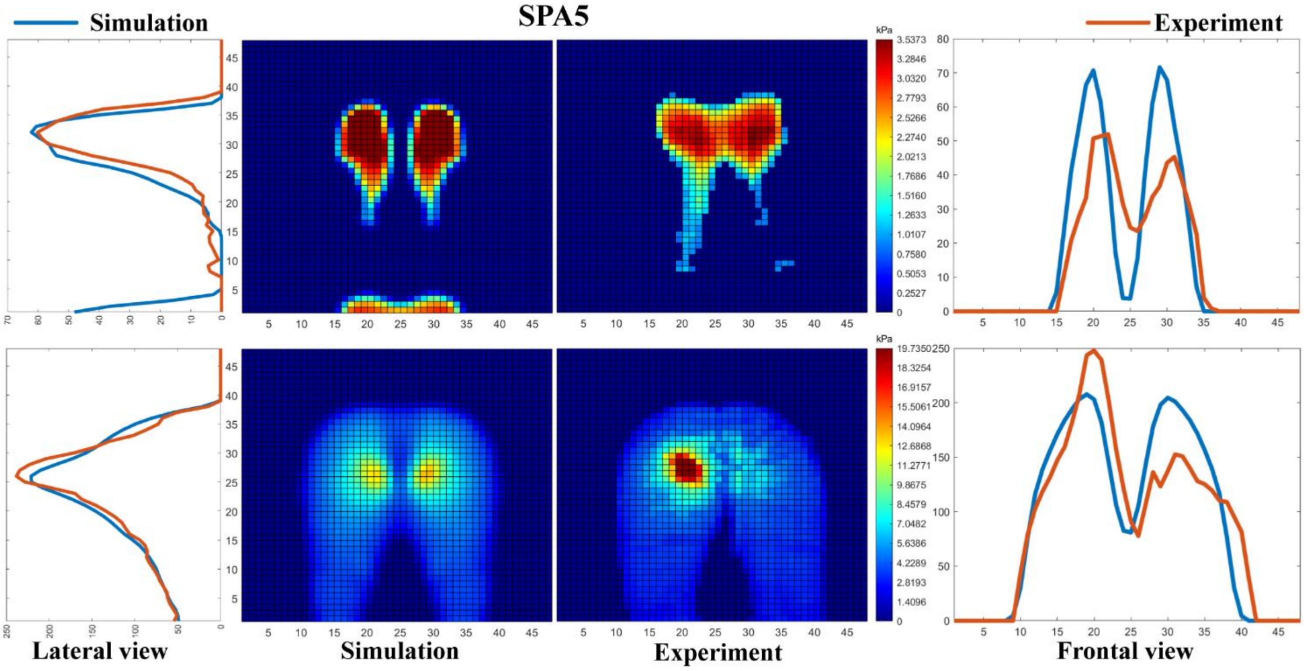

Simulation
SPA5
Experiment
kPa
Lateral view
Simulation
Experiment
Frontal view

(B)

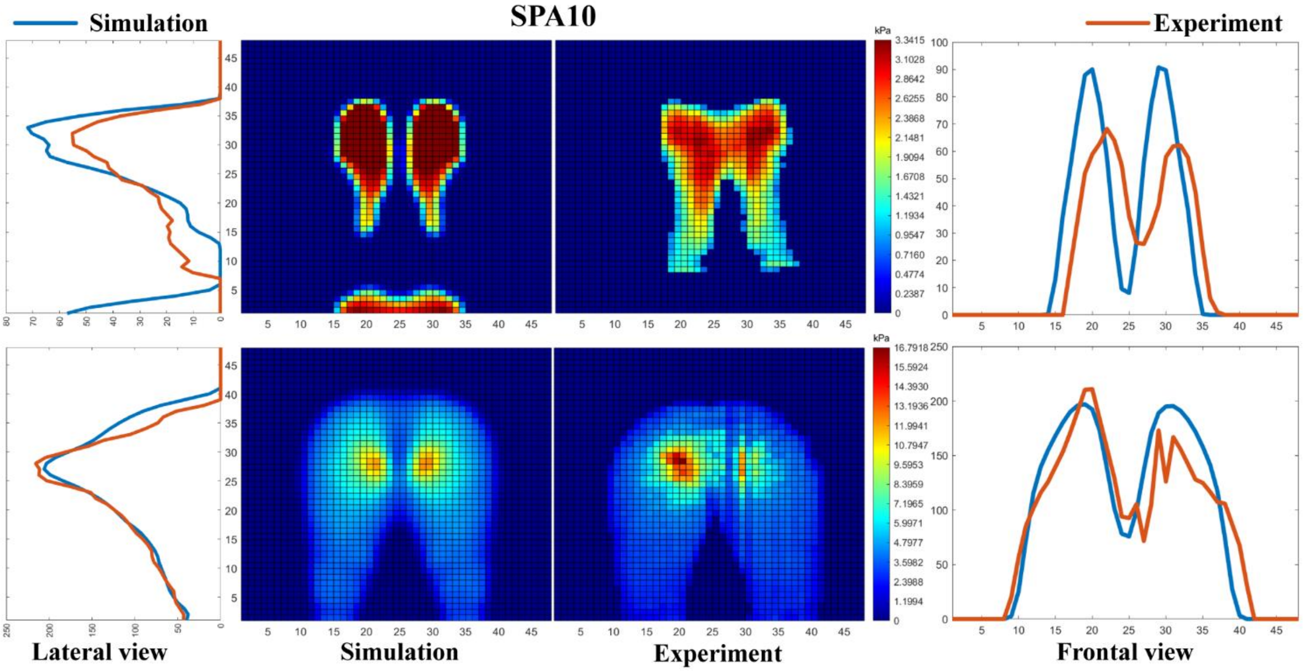

(C)

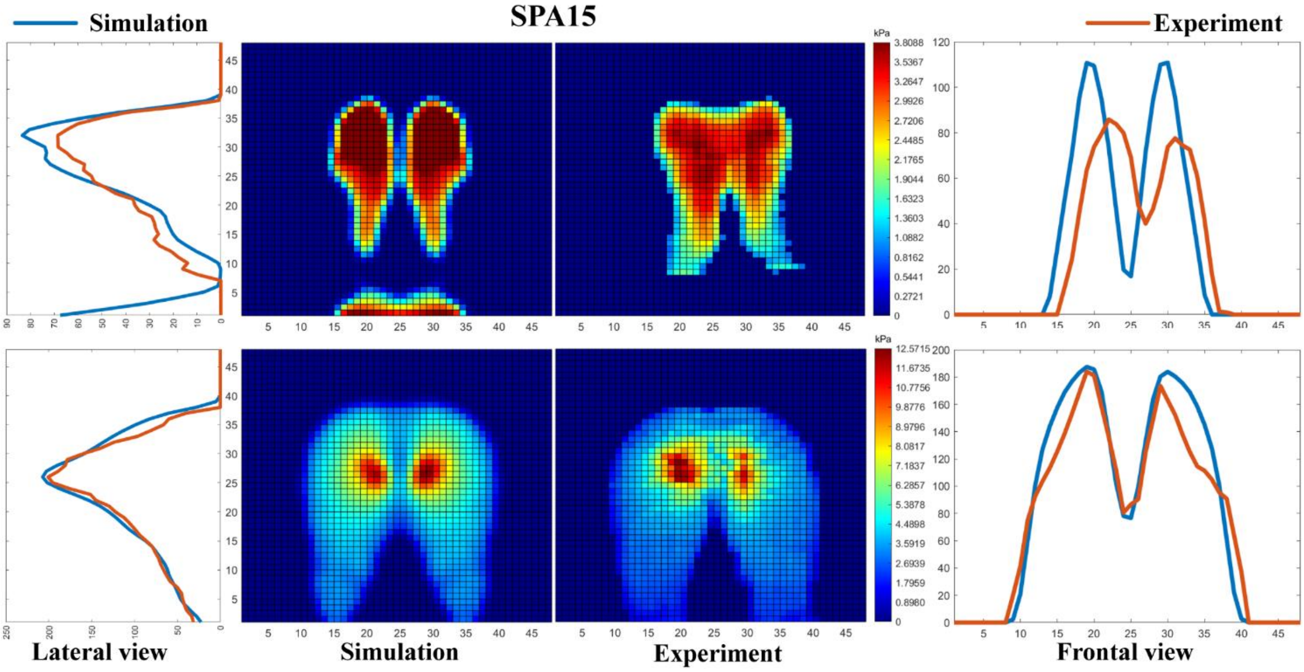

(D)

Figure A4. Experimental and simulated pressure distributions as well as frontal and lateral pressure profiles on the seat pan and backrest for the four seating conditions: (A) SPA0, (B) SPA5, (C) SPA10, (D) SPA15
